# Moxibustion for hypertension: a systematic review

**DOI:** 10.1186/1471-2261-10-33

**Published:** 2010-07-05

**Authors:** Jong-In Kim, Jun-Yong Choi, Hyangsook Lee, Myeong Soo Lee, Edzard Ernst

**Affiliations:** 1 Department of Acupuncture and Moxibustion, College of Oriental Medicine, Kyung Hee University, Seoul, South Korea; 2 Department of Internal Medicine, School of Korean Medicine, Pusan National University, Yangsan, South Korea; 3 Acupuncture and Meridian Science Research Center, College of Oriental Medicine, Kyung Hee University, Seoul, South Korea; 4 Division of Standard Research, Korea Institute of Oriental Medicine, Daejeon, South Korea; 5 Complementary Medicine, Peninsula Medical School, Universities of Exeter & Plymouth, Exeter, UK

## Abstract

**Background:**

Moxibustion is a traditional East Asian medical therapy that uses the heat generated by burning herbal preparations containing *Artemisia vulgaris *to stimulate acupuncture points. The aim of this review was to evaluate previously published clinical evidence for the use of moxibustion as a treatment for hypertension.

**Methods:**

We searched 15 databases without language restrictions from their respective dates of inception until March 2010. We included randomized controlled trials (RCTs) comparing moxibustion to either antihypertensive drugs or no treatment. The risk of bias was assessed for each RCT.

**Results:**

During the course of our search, we identified 519 relevant articles. A total of 4 RCTs met all the inclusion criteria, two of which failed to report favorable effects of moxibustion on blood pressure (BP) compared to the control (antihypertensive drug treatment alone). However, a third RCT showed significant effects of moxibustion as an adjunct treatment to antihypertensive drug therapy for lowering BP compared to antihypertensive drug therapy alone. The fourth RCT included in this review addressed the immediate BP-lowering effects of moxibustion compared to no treatment. None of the included RCTs reported the sequence generation, allocation concealment and evaluator blinding.

**Conclusion:**

There is insufficient evidence to suggest that moxibustion is an effective treatment for hypertension. Rigorously designed trials are warranted to answer the many remaining questions.

## Background

By 2025, the number of adults with hypertension is predicted to be 1.56 billion worldwide [1]. Despite the efforts of conventional healthcare, more than 50% of the patients with high blood pressure (BP) fail to satisfactorily control this condition [2]. One reason is the adverse effects of many antihypertensive drugs, which result in patient noncompliance [3]. Therefore, a substantial proportion of hypertensive patients resort to complementary and alternative medicines to reduce their BP [4,5].

Moxibustion is a traditional East Asian medical intervention that involves the burning of moxa (i.e., *Artemisia vulgaris *or mugwort) directly or indirectly at the acupuncture points. The indications of moxibustion include breech presentation, dysmenorrhea, knee osteoarthritis, diarrhea, asthma, stroke, cancer and hypertension, and so on [6,7]. Unlike the acupuncture stimulation, which involves thrusting or twisting needles, resulting in various biochemical reactions that can have effects throughout the body, moxibustion uses heat stimulation at various temperature levels from mild skin warming to tissue damage from burning. This heat stimulation can yield inflammatory responses and induce vascular changes. It is suggested that around the sites of moxibustion therapy, mediators such as histamine and substance P are released and induce vasodilatation in mice [8]. Such inflammatory responses that affect vascular activity might have some potential to alleviate various cardiovascular diseases, including hypertension. Several animal studies have demonstrated effects on BP following moxibustion treatment. For example, moxibustion given at the acupuncture point of GV26 during halothane anesthesia in non-hypertensive dogs resulted in an increase in arterial BP with a decrease in peripheral resistance [9]. In hypertensive rats, moxibustion given at BL15 decreased the systolic BP (SBP) [10]. Because no review of moxibustion for hypertension is currently available, we conducted a systematic review to evaluate the effectiveness of moxibustion as a treatment for hypertension in human patients.

## Methods

### Data sources

We performed literature searches in the following electronic databases from their respective dates of inception until March 2010: MEDLINE, EMBASE, CINHAL, PsycInfo, The Cochrane Library 2010 (Issue 2), 6 Korean medical databases (Korean Studies Information, DBPIA, Korea Institute of Science and Technology Information, Korea Education and Research Information Service, KoreaMed and Korean National Assembly Library), the Chinese Medical Database (CNKI) and three Japanese medical databases (Japan Science and Technology Information Aggregator, Electronic Science Links Japan, Citation Information by National Institute of Informatics). The search terms used were "moxibustion" and ("blood pressure" or "hypertension") in Korean or Chinese or English. We also manually searched files from our department and several relevant journals (The Journal of Korean Oriental Medicine, The Journal of Korean Acupuncture and Moxibustion Society, The Korean Journal of Meridian and Acupoint, Journal of Korean Oriental Internal Medicine, Journal of Japan Society of Acupuncture and Moxibustion, Focus on Alternative and Complementary Therapies, Forschende Komplementärmedizin und Klassische Naturheilkunde) (Research in Complementary Medicine and Classical Natural Medicine) until March 2010. References were addressed in all the original articles, and reviews were further searched for relevant studies. Dissertations and abstracts during this period were also included.

### Study selection

The inclusion criteria for our review were randomized controlled trials (RCTs) in which moxibustion was administered to human participants with arterial hypertension, which was defined as SBP of 140 mmHg or higher and/or diastolic BP (DBP) of 90 mmHg or higher. All included studies should use an antihypertensive drug or no treatment as a control. Cases where moxibustion was combined with other therapies were excluded from the review. However, subjects taking antihypertensive drugs in combination with moxibustion were included. Studies using interventions of unproven efficacy (e.g., herbs) in the control group were also excluded.

### Extraction of data and quality assessment

All the included articles were read in full. Two independent reviewers (JIK and JYC) extracted the data according to predetermined criteria (Table 1). The Cochrane classification (i.e., randomization, blinding, withdrawals and allocation concealment) was applied to evaluate the risk of bias [11]. In moxibustion trials, practitioner blinding is impossible, and the blinding of the assessors and participants was assessed separately. We defined assessor blinding as an independent person who did not know the participants' allocation who then performed the evaluation of the outcome measures. Differences of opinions between the reviewers were settled through discussion.

**Table 1:** Summary of the randomized clinical studies of moxibustion for hypertension

First author (year)	Mean age or ranges (years) Sex (M/F) Duration of disease (years)	Intervention (regimen)	Control (regimen)	Main outcomes	Intergroup difference	Treated acupuncture points
Jin (2008) [21]	Moxa: 46.5 (19/11); 5.3 Cont: 47.4 (16/14); 6.2	Moxa (30 min, once daily for 10 days, n = 30) Direct (a multifunctional apparatus for moxibustion without smoke), non-suppurative	Antihypertensive drugs (enalapril 10 mg 1 T qd for 10 days, n = 30)	1) Response rate (BP) 2) Response rate (Symptoms: headache, dizziness and insomnia)	1) NS, RR, 1.04 [0.82, 1.32] 2) NS, RR, 1.20 [0.88, 1.64]	GV20, PC6, CV4, ST36, KI1
Zhang (2007) [22]	Moxa: 18-72 (16/14); 2-8 Cont: 35-69 (11/10); 2-16	Moxa (2 hr, 2 times weekly for 1 month, total 10 times, n = 30) Indirect, non-suppurative	Antihypertensive drugs (Various doses of amlodipine from 2.5 mg to 10 mg, qd for 1 month, n = 30)	1) Response rate (BP) 2) Response rate (Symptoms: headache, dizziness and insomnia)	(1) NS, RR, 0.86 [0.63, 1.18] (2) NS, RR, 1.26 [0.94, 1.69]	CV8
Deng (2002) [23]	Moxa: 58.3 (16/14); n.r. Cont: 58.3 (13/17); n.r.	Moxa (n.r. once, n = 30), plus (B) Direct, suppurative	Antihypertensive drugs (Nifedipine sustained release tablet 10 mg bid for 50 days)	1) SBP 2) DBP	1) P < 0.001 (after 30 min) MD, 13.65 [11.54, 15.76]; P < 0.001 (at 50 days) MD, 17.4 [14.14, 20.66] 2) P < 0.001(after 30 min) MD, 8.7 [7.07, 9.28] P < 0.001 (at 50 days) MD, 13.12 [14.14, 20.66]	GB39, ST36
Kim (2005) [24]	Moxa: 61.5 (9/21); n.r. Cont: 66.1 (11/20); n.r.	Moxa (5 times for 2 hours, once, n = 30) Direct, non-suppurative	No treatment (n = 31)	1) SBP 2) DBP	1) NS (after 30 min) MD, -1.67[-5.91, 2.57] P < 0.001(after 60 min) MD, 5.28 [1.14, 9.42] P < 0.001 (after 90 min) MD, 9.35 [5.12, 13.58] P < 0.001 (after 120 min) MD, 10.75 [6.48, 15.02] 2) NS (after 30 min) MD, -0.62 [-2.63, 1.39] NS (after 60 min) MD, 1.06 [-1.13, 3.25] NS (after 90 min) MD, 1.04 [-1.24, 3.32] NS (after 120 min) MD, 2.44 [-0.19, 5.07]	ST36 (Men, left; women, right)

Moxa: moxibustion; nr: not reported; BP: blood pressure; DBP: diastolic blood pressure; SBP: systolic blood pressure; RR: risk ratio; MD: weighted mean difference; NS: no significant difference

### Data synthesis

The mean difference and 95% confidence intervals (CIs) was calculated using the same software for continuous data, and relative risk with 95% CIs was used for dichotomous data in each outcome measure using the Cochrane Collaboration's software (Review Manager version 5.0 for Windows, Copenhagen: The Nordic Cochrane Centre).

## Results

### Study description

**Figure 1:**
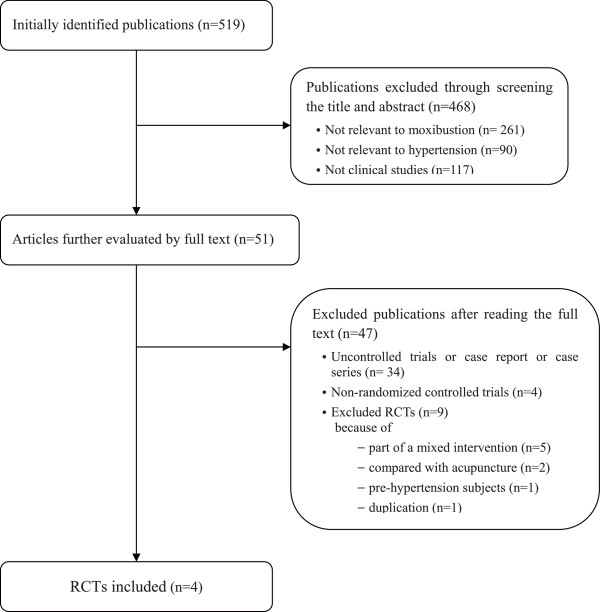
**Flowchart of the trial selection process**. RCT: randomized clinical trial.

We identified 519 articles, of which 515 were excluded (Fig 1). Nine RCTs were among the excluded articles for the following reasons: 5 of the RCTs were excluded because moxibustion was co-administered with other treatments of unproven efficacy [12-16], two RCTs included acupuncture as the control treatment [17,18], one was a duplicate article [19] and the last was an RCT of moxibustion on pre-hypertension subjects [20]. The key data from the 4 RCTs that met our inclusion criteria are listed in Table 1[21-24]. Of these included studies, three were conducted in China [21-23] and one in Korea [24]. All of the included trials had parallel group designs with two groups. Three RCTs [21,23,24] used direct moxibustion, and one [22] employed indirect moxibustion treatment. The main outcome measures were the response rate in two RCTs [21,22], and the SBP or DBP in two RCTs [23,24].

### Risk of bias assessment

None of the included RCTs reported any methods of sequence generation or allocation concealment. One trial mentioned drop-outs and withdrawals [24]. All of the included RCTs failed to report evaluator blinding.

### Details of included trials

Jin *et al*. [21] conducted an RCT to assess the effects of moxibustion on hypertensive patients. Patients were divided randomly into two parallel groups: moxibustion (30 min once daily for 10 days, n = 30) and antihypertensive drugs only (n = 30). The outcome measures included the response rate of the reduction in BP and hypertensive symptoms including headache, dizziness, and insomnia. The response rate of the reduction in BP was defined as the percentage of responders whose DBP decreased more than 10 mmHg or whose SBP decreased more than 30 mmHg. The response rate of the reduction in hypertensive symptoms was defined as the percentage of responders whose hypertensive symptoms in the context of the TCM diagnosis, i.e., headache, dizziness and irritation, decreased from the baseline levels. At the end of the trial, 83% of the patients from the experimental group had a reduced BP, and 80% had improved hypertensive symptoms. The corresponding rates for the control group were 80% and 67%, respectively. There were no significant differences between the two groups in either outcome measure.

Zhang *et al*. [22] tested the effects of indirect moxibustion on the BP and hypertensive symptoms. Sixty patients were randomized into two groups, those receiving moxibustion (2 hr, 2 times weekly for 1 month, n = 30) or antihypertensive drug alone (n = 21). The definition of the response rates in the BP and symptoms were the same as in the Jin *et al*. study [21]. The response rate of the BP was 70% for the moxibustion group (81% for control) and 90% for the improvement of hypertensive symptoms (control: 71%). These parameters failed to yield significant inter-group differences.

Deng *et al*. [23] carried out an RCT to assess the acute antihypertensive effects of moxibustion and the effectiveness of combined moxibustion and antihypertensive drug therapy on SBP and DBP compared to antihypertensive drug therapy alone in 60 hypertensive patients. After the baseline BP measurement, moxibustion was performed once in 30 patients with intentional burn suppuration, while no treatment was administered to the 30 control patients. The BP was assessed again after 30 min. After this, antihypertensive drugs were administered to all 60 patients for 50 days, and the BP was followed up without any further moxibustion treatment. Moxibustion reduced the SBP and DBP significantly after 30 min of treatment (acute effects), and moxibustion combined with drug therapy reduced the SBP and DBP significantly after 50 days of intervention compared to drug therapy only.

A study performed by Kim *et al*. [24] consisted of an RCT evaluating the immediate hypertensive effect of moxibustion compared to no treatment. Sixty-one hypertensive patients were randomized into two groups: one treatment of moxibustion (n = 30) or no treatment (n = 31). The primary outcome was a change in BP at 30, 60, 90 and 120 minutes following treatment. The SBP was significantly reduced in the moxibustion group compared to the control patients at 60, 90 and 120 min after treatment.

We did not perform meta-analysis to avoid the possible bias from pooling of studies with low methodological quality.

## Discussion

By our assessment, the appropriate randomization, allocation concealment and assessor blinding were not reported in any of the 4 RCTs included in our review. Only one study [24] reported the withdrawals and dropouts. This suggests that the risk of bias was high in each of the included studies, potentially leading to false positives. Moreover, all of the studies originated from China and Korea in East Asia. Therefore, additional independent studies in different countries are desirable to determine the generalizability of the results.

In this review, different moxibustion techniques were used in the included RCTs. It is reported that there are more than 50 techniques of moxibustion therapy, and these can be largely classified into direct moxibustion, indirect moxibustion and moxibustion with a moxa stick. Each technique has its own purpose for given situations [25].

Traditionally, direct moxibustion is the main therapy intended to warm and heat the local area by burning a moxa cone. This technique can be applied into two ways, the suppurative (scarring) and non-suppurative (non-scarring) methods. Suppurative moxibustion leaves a burn scar and can yield a very strong inflammatory response, whereas non-suppurative moxibustion is a technique of milder heat stimulation at the local site without any blisters. In the present review, the RCTs of Jin *et al*., Deng *et al*., and Kim *et al*. [21,23,24] administered direct moxibustion therapy, and only the Deng *et al*. trial [23] employed suppurative moxibustion and showed significant efficacy in both the SBP and DBP at 30 min and 50 days after the moxibustion therapy.

In indirect moxibustion therapy, a layer of various herbal medicines, such as ginger, garlic, salt and others, is placed between the moxa cone and skin. The purpose of this technique is the absorption of the therapeutically active components of the herbal medicines into the skin in various conditions combined with heat stimulation by moxibustion. Zhang *et al*. [22] administered indirect moxibustion therapy using mixed powders of various herbal medicines, including *Astragalus membranaceus, Panax notoginseng, Schisandra chinensis *and musk in an umbilicus, without any statistically significant efficacy.

We found no RCTs using moxibustion with a moxa stick in which the moxa stick is placed 2-3 cm from the skin with the intention of mildly warming local sites.

For the optimal moxibustion therapies in future hypertension trials, studies regarding the appropriate moxibustion techniques (direct vs. indirect or other techniques) and the appropriate selection of the herbal cake for indirect moxibustion should be performed. Meanwhile, other techniques should be considered as possible moxibustion therapies for hypertension.

Acupoints are another issue in our review. All of the included RCTs except Zhang *et al*. used ST36. This point showed anti-hypertensive effects in experimental studies when stimulated by acupuncture or electroacupuncture [26,27]. However, all of the included RCTs in our review did not employ any acupoints that had been shown to be antihypertensive by moxibustion stimulation [9,10].

Of the 4 RCTs included here, two studies [21,22] reported response rates by the categorization of the BP change as the primary outcome. This parameter cannot provide the extent of BP reduction or the precise magnitude of effect. Although moxibustion showed non-significant differences from antihypertensive drug treatment with respect to the response rate, we cannot conclude that the BP effect of moxibustion is comparable to that of antihypertensive drugs. This categorical reporting method is common in traditional Chinese medicine research [28]. Thus, additional information on the BP is required to interpret such data.

Two RCTs evaluated the acute effects of moxibustion on BP [23,24]. Of these, one RCT [23] reported a statistically significant BP decrease at 30 minutes after suppurative moxibustion compared to no treatment, while the other study of non-suppurative moxibustion [24] failed to show a significant BP decrease at 30 minutes but showed a significant BP decrease only after 60 minutes. Furthermore, this trial of non-suppurative moxibustion therapy has the limitation of not estimating the long-term effects on BP [24]. The suppurative moxibustion RCT [23] that showed an acute antihypertensive effect also showed significant differences at 50 days after a single treatment, but uncertainty about the effectiveness of a single moxibustion still remains. Also, this RCT [23] showed the superiority of the moxibustion plus antihypertensive drug therapy compared to the antihypertensive drug therapy alone. However, due to the design (A+B versus B), this RCT is unable to demonstrate the specific therapeutic effects of the tested treatment [29]. Two RCTs failed to generate positive effects for moxibustion on BP compared to antihypertensive drug therapy [21,22]. Whether the findings of no difference compared to drug therapy reflect an equivalence of effects is unclear. All of these RCTs have small sample sizes, leading to a higher probability of a type II error. Also, the number of trials and the methodological quality are too low to draw firm conclusions.

All of the RCTs included in our review failed to blind patients because the regimen in the control group (i.e., antihypertensive drugs or no treatment) was easily distinguishable from moxibustion therapy. This suggests that the control groups in these RCTs were inappropriate to determine any specific effect of moxibustion. Recently, a sham device for moxibustion has been developed, making patient-blinding in sham-controlled trials possible [30].

None of the included RCTs reported on adverse events. Furthermore, one RCT, in which intentional suppurative moxibustion therapy was used, had no record of the progress of suppuration and burning in moxibustion-treated participants [23]. Burning has been reported in moxibustion therapy [31], and any minor burning must be monitored. In addition, adverse effects due to smoke can occur during moxibustion and should be monitored with caution [32].

One of the merits of our review is the extensive search strategy without language restriction. However, there is always the possibility of missing studies, not in the least because of a negative publication bias [33-35].

## Conclusion

There are currently only a few trials of moxibustion for the management of hypertension that have been published. Collectively, the existing evidence does not suggest that moxibustion is an effective therapy for this indication. Future studies should be of high quality with particular emphasis on using adequate control interventions and differentiating between specific and non-specific effects.

## Competing interests

The authors declare that they have no competing interests.

## Authors' contributions

JIK, JYC, and MSL conceived of the study design. JYC and JIK searched for and selected the trials and extracted, analyzed and interpreted the data. JIK, JYC and MSL drafted the manuscript. HSL and EE helped with the study design and critically reviewed the manuscript. All authors read and approved the final version of the manuscript.

## Pre-publication history

The pre-publication history for this paper can be accessed here:

http://www.biomedcentral.com/1471-2261/10/33/prepub
